# LC/MS/MS Analysis of N-Terminal Protein Adducts with Improved Sensitivity: A Comparison of Selected Edman Isothiocyanate Reagents

**DOI:** 10.1155/2009/153472

**Published:** 2009-11-04

**Authors:** Per Rydberg, Hans von Stedingk, Jörgen Magnér, Jonas Björklund

**Affiliations:** ^1^Arrhenius Laboratory, Department of Environmental Chemistry, Stockholm University, 106 91 Stockholm, Sweden; ^2^Department of Applied Environmental Science, Stockholm University, 106 91 Stockholm, Sweden; ^3^PerkinElmer Sverige AB, Oxfordhuset, Johanneslundsvägen 2, 194 81 Upplands Väsby, Sweden

## Abstract

This study provides a basis for a new and straightforward method for LC/MS/MS-based screening of N-terminal protein adducts. This procedure is denoted the “FI*R*E procedure” as fluorescein *i*sothiocyanate (FITC) gave superior sensitivity by LC/MS/MS when measuring adducts (*R*) of electrophilic compounds with a modified *E*dman procedure. The principles of the FI*R*E-procedure are that adducts to N-terminal amino acids selectively are detached and measured from of proteins after derivatisation by isothiocyanate Edman reagents. In this study, FITC, 4-N,N-dimethylaminoazobenzene 4′-isothiocyanate (DABITC) and 4-dimethylamino-1-naphthyl isothiocyanate (DNITC) were used to synthesize thiohydantoin analytes from valine and N-methylvaline. The sensitivity by LC/MS/MS was enhanced by up to three orders of magnitude as compared to phenyl isothiocyanate and higher as compared to pentafluorophenyl isothiocyanate. The FITC reagent will enable measurements of low background adduct levels. Synthesized analytes were characterised with, for example, ^1^H NMR, ^13^C NMR, LC/MS/MS, and UV.

## 1. Introduction

Electrophilic chemicals/metabolites presented in vivo can be monitored by measuring the products (adducts) of their reaction with proteins, in particular hemoglobin (Hb) [1–3]. For this purpose methods such as the GC-MS/MS optimized N-alkyl Edman-procedure have been developed for measurement of adducts to N-terminal valine in Hb [4]. 

Although the N-alkyl Edman-procedure has become an established method the method has its limitations, for example, the range of adducts that can be analyzed by GC-MS is limited. Small adducts, for example, ethylene oxide [5], propylene oxide [6], and acrylamide [7, 8], can be quantified at the pmol/g globin level, which is sensitive enough for measurement of background adduct levels (levels found in the general population). However, polar adducts are more difficult to analyse, due to the limitations imposed by the GC separation system prior to MS detection. Some of these obstacles can be solved, for example, by shielding polar groups through additional derivatisation, which for instance was undertaken for measurement of glycidamide adducts in Hb [9]. However, derivatisation of polar groups is both time-consuming and demands the development of new procedures for each specific adduct. Furthermore, adducts of high molecular weight (>500 mass units, mu) and thermolabile adducts are extremely difficult to analyze using the GC-MS based N-alkyl Edman-procedure. 

More recently modifications to the N-alkyl Edman-procedure have been published which instead utilize LC-MS/MS for measurement of adducts after derivatization with phenyl isothiocyanate (PITC) [10, 11] or pentafluorophenyl isothiocyanate (PFPITC) [12] which are the reagents that have been used in this procedure. These modified methods presented by Fennell et al*.* and Ospina et al. [10, 12, 13] have a much higher sample throughput compared with the analysis by GC/MS. The main reason for this higher throughput is that the clean-up procedure is performed on solid phase extraction (SPE) columns, which can be readily automated. Additional benefits are that further derivatization steps of certain analytes (e.g., from polar adducts such as N-[2-hydroxycarbamoylethyl]valine) can be excluded.

Although the existing methods for adduct measurement have been developed and also partly automated, globin [14] and more recently shown the erythrocytes [15] must still be isolated from whole blood and the purification of analytes includes several steps in order to selectively enrich the desired analytes. Furthermore, it is difficult to develop a multianalyte screening procedure with analytes having different polarities as this normally will demand additional purification steps.

Aiming to overcome such problems, this study was designed to evaluate the role of the utilized isothiocyanate reagent regarding mainly the sensitivity when the formed analytes are measured with LC/MS ion trap and LC/MS triple quadrupole instrument. The long-range aim has been to present a more straightforward and general method for adduct measurement. As the utilization of isothiocyanate reagents, for example, FITC leads to formation of analytes with fluorescent properties an alternative route to LC-MS/MS was tested with separation by capillary electrophoresis and spectroscopic measurements.

**Figure 1:**
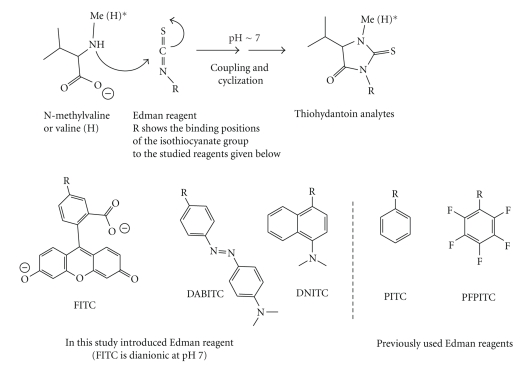
Reactions and Edman reagents compared in this study.

**Figure 2:**
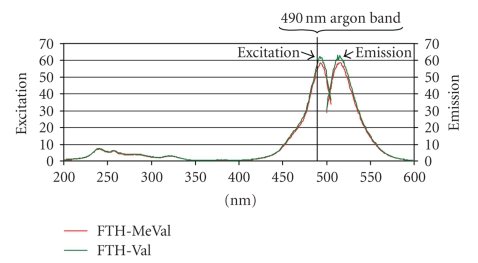
Fluorescence measurements; excitation and emission spectra of the dianions FTH-Val and FTH-MeVal recorded at pH 7.

Three new isothiocyanate reagents were selected and compared with the reagents previously utilized in the N-alkyl Edman procedure. The studied isothiocyanate reagents and reactions are given in Figure 1. The thiohydantoin analytes and the studied reference compounds are given in Table 1. The selected isothiocyanate reagents contained the following ionisable groups, a carboxylic acid group in fluorescein isothiocyanate (FITC) and tertiary amines in both 4-dimethylaminoazobenzene-4′-isothiocyanate (DABITC) and 4-dimethylamino-1-naphthyl isothiocyanate (DNITC). These three reagents were reacted with valine or N-methylvaline, which are models for methylated and normal valyl terminals in Hb, to form methylated and nonmethylated valyl thiohydantoins **1**–**6** (see Figures 1 and 2). These analytes were characterised and relative responses were measured as LOD on two different LC/MS/MS systems under various analytical conditions. The obtained data was then compared with results from analytes formed in the N-alkyl Edman-procedure, mainly PTH-MeVal (**7),** but also with PFPTH-MeVal (**8**) and PFPTH-HOEtVal (**9**). 

## 2. Experimental Procedures

### 2.1. Chemicals

Fluorescein-5-isothiocyanate (isomer I, >90%) and sodium cyanoborohydride (Fluka); 4-N,N-dimethylaminoazobenzene 4′-isothiocyanate (DABITC), 4-dimethylamino-1-naphthyl isothiocyanate (DNITC) (Acros); L-valine (Val), N*-*methyl-D, L-valine (MeVal), 5-isopropyl-3-phenyl-2-thiohydantoin (**7,** PTH-MeVal), and sodium borohydride phosphate buffer (Sigma); ^2^H_3_-acetonitrile (^2^H_3_–ACN, 99.8% ^2^H), (^2^H)chloroform (CDCl_3_, 99.8% ^2^H), deuterium oxide (^2^H_2_O, 99.9% ^2^H), and ^2^H_4_-methanol (99.8% ^2^H) (CIL, Andover, MA) were obtained from the sources indicated. 5-Isopropyl-1-methyl-3-phenyl-2-thiohydantoin (**7**, PTH-MeVal), 5-isopropyl-3-pentafluorophenyl-2-valinethiohydantoin (**8**, PFPTH-MeVal) and 1-hydroxyethyl-5-isopropyl-3-pentafluorophenyl-2-thiohydantoin (**9**, PFPTH-HOEtVal) were synthesized as described earlier [16]. All other chemicals and solvents were of analytical grade. The structures of compounds **1**–**9** and abbreviations are given in Table 1.

### 2.2. NMR Analysis

^1^H and ^13^C NMR spectra were recorded on a JEOL GSX 270 instrument at 270 MHz. All solvents used were fully deuterated; tetramethylsilane (TMS) added as reference was used in chloroform, acetonitrile (ACN), and methanol (MeOH) as internal standard.

### 2.3. TLC Analysis

TLC was performed using silica gel 60 f-254 plates (SiO_2_, Merck) and the spots were visualized with both UV (254 nm) and at long wavelength (378 nm). Melting points were determined using a Büchi 535 instrument. Measurements of pH were carried out using an Orion EA 920 pH-meter equipped with a Ross 8130 glass electrode.

### 2.4. LC/MS/MS Analysis

Two different LC/MS systems were used. *LC/MS-system 1* consisted of a Rheos 4000LC pump (Flux Instruments, Basel, Switzerland) interfaced with an LCQ MS (ThermoQuest, CA, USA). Both electrospray ionization (ESI) and atmospheric pressure-chemical ionization (APCI) were used in a positive and negative mode. 0.1% TFA, 0.1% ammonium acetate, and 0.3 mM aqueous ammonia, were used as pH modifiers. Instrument settings (ESI): Isocratic flow of aqueous buffer/ACN [1 : 1 (v/v)] of 200 **μ**L/min, sheat gas flow 80(N_2_), auxiliary gas flow 10(N_2_), spray voltage 5000 versus −5000 V, capillary temperature 250°C, capillary voltage 10 versus −10 V. Instrument settings (APCI): Isocratic flow of aqueous buffer/ACN [1 : 1 (v/v)] of 500 **μ**L/min, sheat gas flow 70(N_2_), auxiliary gas flow 15(N_2_), capillary temperature 150°C, capillary voltage 5 versus −5 V, evaporation temperature 500°C, discharge current 5 **μ**A. The injection volume was 5 **μ**L and the analytes were dissolved in given solvent mixture at concentrations ranging from 10 **μ**g/mL down to 1 ng/mL, depending on their response and the operating conditions. Determinations in the MS-MS mode were performed by collision-induced dissociation (CID) of the [M+H]^+^ ion. The software Xcalibur (version 1.1) was used for data acquisition, LOD was determined by signal to noise (S/N) of 3, samples were injected in triplicates for each compound and concentration. LC/MS data presented for the studied compounds (**1**–**9**) have been obtained from this system unless otherwise specified.

*LC/MS system 2* consisted of an Agilent 1100 series LC system (Agilent technologies, Santa Clara, CA, USA) interfaced with an API365 triple-quadrupole (MS/MS) detector with a turbo electrospray—interface (ESI) obtained from Sciex, Concord, ON, Canada. An Xterra column C18 (3.5 **μ**m, 1.0 × 100 mm) was used with an isocratic flow at 50 *μ*L/min for determination of relative response (LOD). The injection valve was a six-way standard valve from Valco (VICI International, Switzerland). In positive ion mode the mobile phase was a mixture of H_2_O/ACN [1 : 1 (v/v)] acidified with 0.1% trifluoroacetic acid (TFA). In negative ion mode the mobile phase was water/ACN [1 : 1 (v/v)] buffered with triethylamine acetate (TEAOAc, pH ~ 7). Instrument settings: declustering potential 65 versus −50 V, focusing potential 300 versus −255 V, entrance potential 10 versus −6 V, collision cell exit potential 26.5 versus −15 V, collision energy 54 versus −35.5 V, distance in ion-source 12.5 versus 8.0 cm, deviation in ion-source 7.5 versus 4.0 cm, nebulizer gas (N_2_) set to 15 at both modes, curtain gas (N_2_) set to 15 at both modes, collision gas (N_2_) set to 10 and 2, ion spray voltage 3800 and −3800 V, and vaporizing temperature 250°C at both modes.

Measurements of LOD of compounds **2**,** 4**,** 6**, and** 7** were performed on this system using negative and positive ion modes. After optimizing the collision energy the transition from the precursor ion ([M+H]^+^ or [M − H]^−^) to the product ion with the strongest ion intensity (100%) was used in multiple reaction monitoring mode (MRM). The software Analyst (version 1.4.1) was used for data acquisition, LOD was determined by signal to noise (S/N) of 3, and samples were injected in triplicates for each compound and concentration.

### 2.5. HPLC UV Analysis

Retention times for compounds **1**–**7 **and PTH-Val were determined on a Shimadzu LC-4A (Kyoto, Japan) HPLC system which was connected with a Kromasil LC-18 column (250 × 10 mm) and a Shimadzu SPD-2AS UV (D_2_ lamp) detector. The flow rate was 1.0 mL/min and the mobile phases were A (H_2_O/ACN/TFA, 95/5/0.1), B (H_2_O/ACN/TFA, 20/80/0.1). The gradient profile for compounds **5**–**7 **and PTH-Val was held at 50% B for 5 minutes, then ramped to 100% B in 20 minutes, and held at 100% B for 5 minutes. The gradient profile for compounds **1**–**4 **was held at 100% A for 5 minutes, then ramped to 100% B in 20 minutes, and held at 100% B for 5 minutes. The compounds (100 **μ**M each) were injected to a 50 **μ**L loop. The following wavelengths were used for measurement of the retention times, 268 nm (**1** and **2**), 265 nm (**3**, **4, **PTH-Val, and** 7**), and 260 nm (**5** and **6**). Obtained retention times were as follows: 24.5 minutes (**1**), 26.2 minutes (**2**), 20.3 (**3**), 24.8 minutes (**4**), 18.1 minutes (**5**), 20.9 minutes (**6**), 14.0 minutes (PTH-Val), and 17.7 minutes (**7**).

### 2.6. Capillary Electrophoresis (CE)

Separation was performed on an HP 3D CE column (Agilent, CA, USA) with a five-channel diode array UV detector. A fused silica capillary (i.d. 50 *μ*m, o.d. 375 *μ*m) with a total length of 64 cm and an effective length of 56 cm was used. The separation voltage was +30 kV, resulting in a separation current of 32 *μ*A. The buffer system consisted of 17 mM phosphate buffer (adjusted to pH 7) containing 20 mM SDS and the injection volume was 1 nL.

### 2.7. Fluorescence Measurements

Excitation and emission spectra were recorded in water : acetonitrile solutions (9:1) buffered at pH 1, 3, 5, 7, and 9 with 0.1 M standard buffers (exact composition presented for shown data) using a Shimadzu RF5000 fluorescence spectrophotometer. Emission was scanned from 10 nm above the excitation wavelength, locked at the optimal absorbance wavelength determined from the UV absorbance measurements described above, to 600 nm. Excitation was then scanned from 200 nm to 10 nm below the emission wavelength locked at the optimal wavelength obtained during the emission scan. Fluoranthene was used as reference.

### 2.8. Synthesis of Reference Compounds/Analytes

#### 2.8.1. Synthesis of Fluorescein, 5-(4-isopropyl-2-thioxo-imidazolidin-5-one) (**1**, FTH-Val)

**Figure 3:**
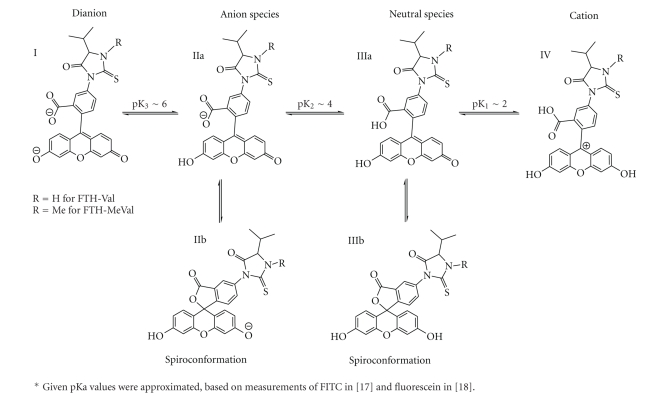
Chemical forms the FTH-analytes in aqueous solution*.

From a stock solution of 0.50 M L-valine (20 mmol in 40 mL 0.25 M KOH), a 5.0-mL aliquot (2.5 mmol Val) was heated to 45°C and then reacted with FITC (0.50 mmol, 199 mg, dissolved in 6 mL dioxane/H_2_O, 10:1). The reaction was interrupted after 90 minutes when all FITC had been consumed (as seen on TLC) and the intermediate, fluorescein thiocarbamoyl-valinate, was converted to the ring-closed FTH-Val by addition of 1 mL (12 mmol) hydrochloric acid (HCl). For convenience, this reaction was stopped after 14 hours at 45°C. The solvent was evaporated under vacuum and the dry solid remaining was dissolved in a two-phase solvent system consisting of water : ethyl acetate (EtOAc) (1 : 1, total volume 60 mL). The EtOAc phase was extracted with H_2_O (15 mL × 2). The combined aqueous phases were then extracted with EtOAc (20 mL) and combined with the first EtOAc phase, which was finally extracted with H_2_O (2 × 15 mL). The organic phase was dried with Na_2_SO_4_. After filtration and evaporation the residue was dissolved in EtOAc/MeOH (4 : 1, v/v) and purified by column chromatography (SiO_2_, 25 × 3 cm) eluted with EtOAc/MeOH (4 : 1, v/v). The fractions containing the products were combined, dried by evaporation and crystallized from ethanol/H_2_O (1 : 1, v/v) to yield 167 mg (68%) of the desired product. Analysis: m.p. 232.5–234.5°C, Rf-TLC 0.65 (EtOAc/MeOH, 4 : 1, v/v), UV (H_2_O/ACN, 1 : 1, v/v) *λ*_max _ = 268 nm, *ε*_268_ = 21500. Fluorescence measurements (0.10 **μ**M of comp. **1** in H_2_O/ACN, 9:1, v/v, buffered with at pH 7 0.1 M phosphate buffer pH 7) ex. wavelength 492 nm, em. wavelength 515 nm (max) (see Figure 2). ^1^H NMR (^2^H_6_-acetone), 25°C) *δ*1.08, 1.18 [d+d, 3+3H, *J* = 6.9, 6.6 Hz, CH_3_(*γ*, *γ*′)], 2.38 [m, 1H, *J* = 3.8, 6.9 Hz, CH(*β*)], 2.86 [s, 1H, not assigned] 4.4 [d, 1H, *J* = 3.8 Hz, CH(*α*)], 6.66–6.77 [6H, m with the most pronounced peak at 6.71, xanthene-H], 7.39, 7.42 [d+d, 1H, *J* = 0.55, 8.2 Hz, C_7_–H] 7.74, 7.77 [d+d, 1H, *J* = 1.9, 8.2 Hz, C_6_–H], 7.96 [d, 1H, *J* = 1.6 Hz, C_4_–H] 9,0 [s, 2H, xanthene-(OH)_2_] 9,5 [s, 1H, N–H]. ^13^C NMR (^2^H_6_-acetone/CD_3_OD, 2 : 1, v/v, 25°C) *δ* 17.0, 18.9 [CH_3_(*γ*,* γ*′)], 32.5 [CH(*β*)], 66.3 [CH(*α*)], 103.7, 113.8, 125.9, 126.1, 130.5, 154.0, 161.3 [xanthene carbons], 136.5, 136.9 [C_2_+C_5_], 169.9 [COO], 175.2 [CO], 184.6 [CS]. The chemical shifts of five of the carbon atoms are not given (impossible to separate from the background noise). However, the presented carbons were in accordance with the predicted shifts of compound **1** in its assumed spiroconformation (cf. IIIb in Figure 3). Analysis by LC/MS, ESI, positive ion mode gave *m/z* 489.2 [M+H]^+^, ESI negative ion mode gave *m/z* 487.2 [M − H]^−^. Analysis by LC/MS/MS (ESI+, collision energy 40 V), product ions from *m/z* 489.2 (rel. int. 29%): *m/z* 446.1 [M+H^+^ – 43 (-isPr) 16%], 390.3 [M+H^+^ – 99 (FITC+H^+^), 100%], the same ions were used and formed when measuring LOD by LC/MS/MS APCI+ (collision energy 40 V). LC/MS/MS (ESI-, collision energy −30 V), product ions from *m/z* 487.2 (rel. int. 3%): 443.2 [M^−^ − H – 44 (-isPr+H)^−^, 100%], *m/z* 384.2 [(M − H – 103)^−^, 6%], 372.1 [M^−^–H –115 (fluorescein isocyanate anion – H), 17%], 288.5 [(M^−^ − H – 202), 15%].

#### 2.8.2. Synthesis of Fluorescein, 5-(4-isopropyl-3-methyl-2-thioxo-imidazolidin-5-one) (**2**, FTH-MeVal)

From a stock solution of 0.500 M MeVal (20 mmol in 40 mL 0.25 M KOH), a 5.0-mL aliquot (2.5 mmol MeVal) was heated to 45°C and then reacted with FITC (0.50 mmol, 199 mg) dissolved in 6 mL dioxane/H_2_O (10:1). This reaction was monitored by TLC and after 90 minutes all of the FITC had been consumed. In order to extract the product formed, the solution containing FTH-MeVal was acidified with concentrated HCl (1 mL, 12 mmol) and extracted as described above. After evaporation to dryness the product was purified by column chromatography and crystallized as described above to yield 194 mg (77%) of the desired product. Analysis: m.p. 213.5–217°C, Rf-TLC 0.64 (EtOAc/MeOH, 4 : 1, v/v), UV (ACN/H_2_O, 1 : 1, v/v) *λ*_max _ = 268 nm, *ε*_268_ = 17 500. Fluorescence measurements (0.10 **μ**M of comp. **2** in H_2_O/ACN, 9:1, v/v, buffered with 0.1 M phosphate buffer pH 7) ex. wavelength 492 nm, em. wavelength 515 nm (max) (see Figure 2). ^1^H NMR (^2^H_6_-acetone, 25°C) *δ*1.01, 1.25 [d+d, 3+3H, *J* = 6.9 Hz, CH_3_(*γ*,* γ*′)], 2.57 [m, 1H, *J* = 3.6, 6.9 Hz, CH(*β*)], 2.87 [s, 1H, not assigned] 3.4 [s, 3H, N–CH_3_], 4.4 [d, 1H, *J* = 3.0 Hz, CH(*α*)], 6.65–6.77 [6H, m with the most pronounced peak at 6.71, xanthene-H], 7.39, 7.42 [d+d, 1H, *J* = 0.55, 8.2 Hz, C_7_–H] 7.72, 7.75 [d+d, 1H, *J* = 1.9, 8.2 Hz, C_6_–H], 7.93 [d, 1H, *J* = 1.9 Hz, C_4_–H] 9,0 [s, 2H, xanthene-(OH)_2_]. ^13^C NMR (acetone, 25°C) *δ*16.3, 17.5 [CH_3_(*γ*,* γ*′)], 30.3 [CH(*β*)], 33.1 [N–CH_3_], 69.3 [CH(*α*)], 103.5, 113.5, 125.4, 125.7, 130.2, 153.4, 153.8, 160.5 [xanthene carbons] 136.6 [C_2_ or C_5_], 168.7 [COO], 172.7 [CO], 182.7 [CS]. The chemical shifts of six of the carbon atoms are not given (impossible to separate from the background noise); however, the presented shifts were in accordance to the predicted shifts for compound **2** in its assumed spiro-conformation (cf. IIIb in Figure 3). Analysis by LC/MS, ESI, positive ion mode gave *m/z* 503.3 [M+H]^+^, ESI negative ion mode gave *m/z* 501.2 [M − H]^−^. Analysis by LC/MS/MS (ESI+, collision energy 40 V), product ions from *m/z* 503.3 (rel. int. 47%): *m/z* 460.1 [M+H^+^ – 43 (-isPr), 100%], 390.3 [M+H^+^ −112 (FITC+H^+^), 19%,]. LC/MS/MS (ESI-, collision energy −35 V), product ions from *m/z* 501.2 (rel. int. 0%): *m/z* 457.3 [M^−^ − H – 44 (-isPr+H), 100%]. LC/MS/MS (APCI+, collision energy 40 V), product ions from *m/z* 503.2 (rel. int. 10%): *m/z* 460.1 [M+H^+^ – 43 (-isPr), 100%], 390.3 [M+H^+^ −112 (FITC+H)^+^, 30%]. LC/MS/MS (APCI-, collision energy −28 V), product ions from *m/z* 501.2 (rel. int. 10%): *m/z* 457.3 [M^−^ − H – 44 (-isPr –H), 100%].

#### 2.8.3. Synthesis of 3-[4-(4-dimethylamino-phenylazo)-phenyl]-5-isopropyl-2-thioxo-imidazolidin-4-one (**3**, DABTH-Val)

From a stock solution of 0.500 M L-valine (20 mmol in 40 mL 0.25 M KOH), a 5.0-mL aliquot (2.5 mmol Val) was heated to 60°C and mixed with 0.10 M DABITC (5.0 mL, 0.50 mmol) in dioxane. In order to obtain a homogeneous solution, an additional 3.5 mL dioxane and 2.5 mL H_2_O was added. The reaction was monitored by TLC (EtOAc/MeOH, 4 : 1, v/v) and after 30 minutes the reagent, DABITC, had been consumed. Concentrated HCl (1.0 mL, 12 mmol was added in order to convert the 4-N,N-dimethylaminoazobenzene 4′-thiocarbamoyl-valine formed to the corresponding ring-closed DABTH-Val (**3**). This reaction was also monitored by TLC (toluene/EtOAc, 2 : 1, v/v; spots developed with both UV and long wavelength light) and found to be complete after 30 minutes at 60°C. The solution was extracted with chloroform (25 mL) and H_2_O (25 mL), the aqueous phase thus obtained neutralized with KHCO_3_, and the product extracted from this phase with chloroform (2 × 25 mL). This chloroform phase was subsequently extracted with H_2_O (25 mL) and was then dried with Na_2_SO_4_. After filtration and evaporation, the orange-coloured solid obtained was crystallized from dichloromethane/hexane (2 : 1) to yield 132 mg (69%) of the described product. Analysis: m.p. 225°C, Rf-TLC 0.73 (EtOAc/MeOH, 4 : 1, v/v), UV (ACN/H_2_O, 1 : 1, v/v) *λ*_max _ = 265 nm, *ε*_265_ = 28 000. ^1^H NMR (CDCl_3_, 25°C) *δ*1.06, 1.15 [d+d, 3+3H, *J* = 6.9, 7.1 Hz, CH_3_(*γ*,* γ*′)], 2.39 [m, 1H, *J* = 3.3, 6.9, 7.1 Hz, CH(*β*)], 3.09 [s, 6H, N(CH_3_)_2_] 4.18 [d, 1H, *J* = 3.3, CH(*α*)], 6.75, 6.77 [d+d, 2H, azobenzene C_6_–H, C_7_–H] 7.39, 7.41 [d+d, 2H, azobenzene C_5_–H, C_8_–H] 7.88, 7.90, 7.94, 7.96 [4d, 4H, azobenzene C_1_–H–C_4_–H]. Analysis by LC/MS, ESI, positive ion mode gave *m/z* 382.2 [M+H]^+^, ESI negative ion mode gave *m/z* 380.2 [M − H]^−^. Analysis by LC/MS/MS (ESI+, collision energy 40 V), product ions from *m/z* 382.2 (rel. int. 2%): *m/z* 339.1 [(M+H^+^ – 43 (-isPr), 2%], 283.1 [(M+H – 99, DABITC+H)^+^, 19%,], 232.9 [(M+H^+^ – 149 (-PhNMe_2_), 25%], 205.0 [(M+H^+^ – 177), 100%], LC/MS/MS (ESI-, collision energy −35 V), product ions from *m/z* 380.2 (rel. int. 2%): *m/z* 337.0 [M^−^ − H – 43 (-isPr), 4%], 264.3 [M^−^ − H – 116, 100%].

#### 2.8.4. Synthesis of 3-[4-(4-dimethylamino-phenylazo)-phenyl]-5-isopropyl-1-methyl-2-thioxo-imidazolidin-4-one (**4**, DABTH-MeVal)

From a stock solution of 0.50 M MeVal (20 mmol in 40 mL 0.25 M KOH), a 5.0-mL aliquot (2.5 mmol MeVal) was heated to 60°C and mixed with 0.10 M DABITC (5.0 mL, 0.50 mmol) in dioxane. In order to obtain a homogeneous solution another 3.5 mL dioxane and 2.5 mL H_2_O were added. This reaction mixture became inhomogeneous in contrast to the mixture containing Val, indicating that the DABTH-MeVal formed precipitated. The reaction was monitored by TLC (SiO_2_, toluene/EtOAc, 2 : 1, v/v; spots developed with both UV and long wavelength light) and after 30 minutes all the DABITC had been consumed. The subsequent extraction was performed as described above. The product crystallized from dichloromethane/hexane (1:4, v/v) to yield 189 mg (99%) of the desired product. Analysis: m.p. 164.5–169°C, Rf-TLC 0.73 (SiO_2_; EtOAc/MeOH, 4 : 1, v/v), UV (ACN/H_2_O 1 : 1, v/v) *λ*_max _ = 265 nm, *ε*_265_ = 26 000, ^1^H NMR (CDCl_3_, 25°C): *δ*1.05, 1.25 [d+d, 3+3H, *J* = 6.9, 7.1 Hz, CH_3_(*γ*,* γ*′)], 2.47 [m, 1H, *J* = 3.3, 6.9, 7.1 Hz, CH(*β*)], 3.09 [s, 6H, N(CH_3_)_2_] 3.4 [s, 3H, N–CH_3_], 4.4 [d, 1H, *J* = 3.3 Hz, CH(*α*)], 6.74, 6.77 [d+d, 2H, azobenzene C_6_–H, C_7_–H] 7.37, 7.40 [d+d, 2H, azobenzene C_5_–H, C_8_–H] 7.87, 7.90, 7.92, 7.95 [4d, 4H, azobenzene C_1_–H–C_4_–H]. Analysis by LC/MS, ESI, positive ion mode gave *m/z* 396.2 [M+H]^+^, ESI negative ion mode gave *m/z* 394.3 [M–H]^−^. Analysis by LC/MS/MS (ESI+, collision energy 40 V), product ions from *m/z* 396.2 (rel. int. 3%): *m/z* 353.1 [M+H^+^ – 43 (-isPr), 7%], 246.9 [M+H^+^ – 149 (DABITC+H^+^), 35%,], 219.0 [M+H^+^ – 177 (-PhNMe_2_), 100%]. The same ions were used and formed when measuring LOD by LC/MS/MS APCI+ (collision energy 38 V). 178.1 [(M+H^+^ – 218)^+^, 8%] LC/MS/MS (ESI-, collision energy −40 V), product ions from *m/z* 394.3 (rel. int. 2%): *m/z* 379.3 [M^−^ − H – 15 (–Me), 100%], 366.2 [M^−^ − H – 28 (–CO), 6%], 239.2 [M^−^ − H −155 (N,N-dimethyl-4,4′-azodianiline – H)^−^, 26%].

#### 2.8.5. Synthesis of 3-(4-dimethylamino-naphthalene-1-yl)-5-isopropyl-2-thioxo-imidazolidin-4-one (**5**, DNTH-Val)

L-Valine (804 mg = 6.86 mmol) was alkalized with KOH (3.43 mmol) and dissolved in 0.5 M KHCO_3_ (3 mL) and dioxane (3 mL). This solution was heated to 45°C by magnetic stirring and DNITC (88 mg, 0.38 mmol) dissolved in dioxane (2 mL) was added to the suspension. The reaction was monitored by TLC (SiO_2_; EtOAc/MeOH, 4 : 1, v/v; pure toluene and toluene/EtOAc, 2 : 1, v/v) using a reference without valine. After 60 minutes the reaction was complete, since no DNITC could be detected by TLC and the carbamoylated product formed, that is, the N-[4-(N-dimethylamino)-1-naphthalenyl]-thiocarbamoyl-N′-valinate gave fluorescence at long wavelength under the UV lamp (seen as a tailing spot on TLC eluted with EtOAc/MeOH, 4 : 1, v/v). The reaction mixture was acidified with 3 mL concentrated HCl (36 mmol). After 2 hours at 45°C, the reaction mixture neutralized by adding solid KHCO_3_ until no more carbon dioxide was released. The reaction mixture was diluted with water (100 mL) and the product purified by extraction with CHCl_3_ (125 mL). The organic phase was dried with Na_2_SO_4_ and filtered, and the solvent removed by evaporation to yield 126 mg (96%) of the desired product as a white solid. Analysis: m.p. 107.5–110°C, Rf-TLC 0.73 (EtOAc/MeOH, 4 : 1, v/v), UV (ACN/H_2_O, 1 : 1, v/v) *λ*_max _ = 260 nm, *ε*_260_ = 21 000, ^1^H NMR (^2^H_6_-acetone, 25°C) *δ*1.10, 1.25 [d+d, 3+3H, *J* = 6.9, 7.1 Hz, CH_3_(*γ*,* γ*′) assigned to the thiohydantoin], 1.17–1.21 [d+d, 3+3H, *J* = 6.9 Hz, CH_3_(*γ*,* γ*′) assigned to the thiocarbamoyl], 2.40 [m, 1H, *J* = 3.8, 6.9 Hz, CH(*β*)], 2.92 [s, 6H, N(CH_3_)_2_] 4.45, 4.56 [d+d, 1H, *J* = 1.65, 3.85 Hz, CH(*α*)], 7,17–8,32 [m, 6H, naphthalene-H]. ^13^C NMR (^2^H_6_-acetone, 25°C) *δ*16.8, 17.4; 18.7, 19.0 [2x CH_3_(*γ*,* γ*′)], 31.66; 32.0 [2x CH(*β*)], 45.4 [N-(CH_3_)_2_], 65.7; 66.1 [2x CH(*α*)], 114–153 [naphthalene], 174.9 [CO], 185.8; 185.9 [2x CS]. This molecule was found to be partly hydrolyzed to the corresponding ring-opened thiocarbamoyl compound. All shifts obtained are presented, but not assigned to the individual compounds. Analysis by LC/MS, ESI, positive ion mode gave *m/z* 328.2 [M+H]^+^, ESI negative ion mode gave *m/z* 326.3 [M − H]^−^. Analysis by LC/MS/MS (ESI+, collision energy 35 V), product ions from *m/z* 328.2 (rel. int. 1%): *m/z* 313.0 [M+H^+^ – 15 (–Me), 100%], 229.0 [M+H^+^ – 99 (DNITC+H^+^), 13%,]. LC/MS/MS (ESI-, collision energy −35 V), product ions from *m/z* 326.1 (rel. int. 2%) *m/z* 267.3 [M^−^ − H – 59, 100%].

#### 2.8.6. Synthesis of 3-(4-dimethylamino-naphthalene-1-yl)-5-isopropyl-1-methyl-2-thioxo-imidazolidin-4-one (**6**, DNTH-MeVal)

MeVal (93.0 mg, 0.708 mmol) was alkalized with 1 M KOH (400 **μ**L, 0.4 mmol) and dissolved in 0.5 M KHCO_3_ (3 mL) and dioxane (2 mL). This solution was heated to 45°C during magnetic stirring and 4-dimethylnaphthylisothiocyanate (DNITC, 91.3 mg, 0.40 mmol) dissolved in dioxane (2 mL) was added. The reaction was monitored by TLC (SiO_2_; toluene and toluene/EtOAc, 2 : 1, v/v) using a reference without MeVal. After 60 minutes, the reagent DNITC had been consumed and following TLC elution with toluene/EtOAc, the analyte formed could be visualized by its fluorescence at long wavelength under the UV lamp. The reaction mixture was extracted with toluene and purified by column chromatography (SiO_2_) eluted with toluene/EtOAc (2 : 1, v/v) to yield 130 mg (96%) of the desired product. This product was crystallized from ethanol/H_2_O (1 : 1) to yield white crystals (100 mg, 73%) with the following characteristics: m.p. 157–159°C, Rf-TLC 0.72 (EtOAc : MeOH, 4 : 1, v/v), UV (ACN/H_2_O 1 : 1, v/v) *λ*_max _ = 260 nm, *ε*_260_ = 21 000, ^1^H NMR (^2^H_6_-acetone, 25°C) *δ*1.04, 1.16 [d+d, 3+3H, *J* = 6.9, 7.1 Hz, CH_3_(*γ*, *γ*′) assigned to the thiohydantoin], 1.24, 1.28 [d+d, 3+3H, *J* = 6.9 Hz, CH_3 _(*γ*, *γ*′′) assigned to the thiocarbamoyl], 2.59 [m, 1H, *J* = 3.3, 6.9, 7.1 Hz, CH(*β*)], 2.92 [s, 6H, N(CH_3_)_2_] 3.41, [d, 3H, *J* = 1.65, Hz, N–CH_3_] 4.38, 4.50 [d+d, 1H, *J* = 3.0, 3.3 Hz, CH(*α*)], 7,15–8,31 [m, 6H, naphthalene-H]. ^13^C NMR (^2^H_6_-acetone, 25°C) *δ*16.3, 17.1; 17.5, 17.8 [2x CH_3_(*γ*, *γ*′′)], 30.3 [CH(*β*)], 33.0; 33.1 [2x N-CH_3_], 45.4 [N-(CH_3_)_2_], *δ*69.1; 69.4 [2x CH(*α*)], 114–153 [2x naphthalene], 173.5 [CO], 184.3; 184.4 [2x CS]. This molecule was found to be partially hydrolyzed to the corresponding ring-opened thiocarbamoyl compound. All shifts obtained are presented, but not assigned to individual compounds. Analysis by LC-MS, ESI, positive ion mode gave *m/z* 342.2 [M+H]^+^, ESI negative ion mode gave *m/z* 340.2 [M − H]^−^. Analysis by LC-MS/MS (ESI+, collision energy 35 V), product ions from *m/z* 342.2 (rel. int. 2%): *m/z* 327.1 [M+H^+^ – 15 (–Me), 100%], 229.2 [M+H^+^ – 99 (DNITC+H^+^), 66%]. The transition *m/z *342.2 → 327.1 was used when measuring LOD by LC-MS/MS APCI+ (collision energy 32 V). LC-MS/MS (ESI-, collision energy −35 V), product ions from *m/z* 340.2 (rel. int. 17%): *m/z* 325.2 [M^−^ − H – 15 (–Me), 100%].

### 2.9. LC-MS Characterization of 5-isopropyl-1-methyl-3-phenyl-2-thiohydantoin (PTH-MeVal, **7**), 5-isopropyl-3-pentafluorophenyl-2-thiohydantoin (PFPTH-MeVal, **8**) and 1-hydroxyethyl-5-isopropyl-3-pentafluorophenyl-2-thiohydantoin (PFPTH-HOEtVal, **9**)

Analysis by LC-MS, ESI (positive ion mode) gave *m/z* 249.1 (M+H^+^) for comp. **7**, *m/z* 349.1, *m/z* 339 for comp. **8**, and *m/z* 369.1 for comp. **9**. LC-MS, ESI negative ion mode gave *m/z* 247 (M^−^ − H) for comp. **7**, *m/z* 337.0 (M^−^ − H) for comp. **8**, and *m/z* 367.1 (M^−^ − H) for comp. **9**. Analysis of comp. **7 **by LC-MS/MS (ESI+, collision energy 35 V), product ions from *m/z* 249.1 (rel. int. 2.5%): *m/z* 207.1 [M+H^+^ – 42 (-IsPr–H), 100%], 176.1 [M+H^+^ −73 (–CSNMe+H)^+^, 55%,], LC-MS/MS (ESI-, collision energy −40 V), product ions from *m/z* 247.1 (rel. int. 0%): *m/z* 232.1[M^−^ − H – 15 (–Me), 100%). The transaction *m/z* 249.1 → 207.2 were used when measuring LOD by LC-MS/MS APCI+ (collision energy 33 V). Analysis of comp. **8 **by LC-MS/MS (APCI+ collision energy 40 V), product ions from *m/z* 339.2 (rel. int. 6%): *m/z* 311.0 [M–H^+^ – 28 (–CO), 100%], 266.0 [M–H^+^ – 73 (–CSNMe), 95%]. LC-MS/MS (APCI-, collision energy −30 V), product ions from *m/z* 337.0 (rel. int. 10%): *m/z* 317.2 [M^−^ − H – 20 (–HF), 100%], 289.1 [M^−^ − H – 48 (–CS), 50%]. Analysis of comp. **9 **by LC-MS/MS (ESI+) gave no product ions from *m/z* 369.1. LC-MS/MS (ESI-, collision energy −25 V), product ions from *m/z* 367.1 (rel. int. 1%): *m/z* 347.1 [M^−^ − H – 20 (–HF), 100%] and *m/z* 287.2 [M^−^ − H – 80, 5%]. LC-MS/MS (APCI+ collision energy 28 V), product ions from *m/z* 369.1 (rel. int. 10%): *m/z* 351.0 [M − H^+^ – 18 (–H_2_O), 70%], 223.1 [M − H^+^ – 46 (–CH_2_CH_2_OH+H), 100%]. LC-MS/MS (APCI-, collision energy −25 V), product ions from *m/z* 367.1 (rel. int. 2%): *m/z* 347.1 [M^−^ − H – 20 (–HF), 100%].

## 3. Results

### 3.1. General Experimental Setup

In order to investigate if the original N-alkyl Edman-procedure could be better adapted for measurement by LC-based analytical systems, fluorescent and ionisable isothiocyanate reagents were explored. The selected reagents were reacted with N-methylvaline and valine which are models for methylated and normal valyl terminals in Hb, to form the corresponding methylated and nonmethylated valyl thiohydantoin (compounds **1**–**6**, Table 1). These compounds were purified and compared with regards to LOD on LC/MS/MS with the phenylthiohydantoin of methylvaline (PTH-MeVal, **7**) and, in few experiments, with the pentafluorophenylthiohydantoins of methylvaline and 2-hydroxyethylvaline (PFPTH-MeVal, **8** and PFPTH-HOEtVal, **9**, resp.). Compound **7** is formed from phenyl isothiocyanate (PITC) and compounds **8** and **9** are formed from pentafluorophenyl isothiocyanate (PFPITC); both reagents are utilized in the N-alkyl Edman-procedure (see Table 1).

The analytes were analysed by ^1^H NMR (compounds **1**–**6**), ^13^C NMR (**1**, **2**, **5**, and **6**), LC/MS/MS (**1**–**9**), CE-DAD (**1**-**2**), and UV/vis spectroscopy (**1**–**7**). Compounds **1**-**2** were also characterized by fluorescence spectroscopy. In general, for all these measurements and determinations, the pH was below or above pKa for the ionisable group of each respective analyte (**1**–**6**).

### 3.2. Coupling and Cyclisation Reaction

TLC was used to follow the coupling reaction (compounds **1**–**6)**. In general terms, the coupling/cyclisation reaction was complete within 15 minutes when reacting FITC, DABITC, and DNITC with N-methylvaline (**2**, **4**, and **6**), whereas the cyclisation reaction for nonmethylated valine analytes (**1**, **3**, and **5**) took place first after an additional acidification step. This is in accordance with the traditional Edman degradation procedure [17] and the previous studies [15, 17]. The coupling/cyclisation reaction was also studied using model peptides, N-methylvalylleucylanilide, and ValLeuSer derivatised with FITC. The results were in accordance with the studies on model amino acids; FTH-MeVal was formed directly during the conditions for the coupling reaction (pH ~ 8) while FTH-Val was formed first after an additional acidification step. All reactions could easily be followed on TLC as the spots were visible to the naked eye. Intermediates and products formed from FITC, DABITC, and DNITC could also be visualized with the wavelengths 366 and 245 nm, respectively.

### 3.3. NMR

^1^H NMR was used for structural determination and for studies on purities of analytes (**1**–**6**). It could also be established that the analytes (**1**–**6**) were in the cyclic, thiohydantoin, conformation, which was seen by the well separated shifts for the protons assigned to the *γ*, *γ*′-methyls (dd) in the valine spin residue. For ring-open analytes, in the thiocarbamoyl conformation, the *γ*, *γ*′-methyls are only slightly separated (c.f. [18]).

### 3.4. LC Separation of Standards

Full baseline separation of the methylated and nonmodified tiohydantoins (compounds **1**–**7** and PTH-Val) was achieved with a reversed phase C18 column, using gradients of water and acetonitrile acidified with 0.1% TFA.

### 3.5. MS-Analysis, Comparison of Relative Response (LOD)

**Figure 4:**
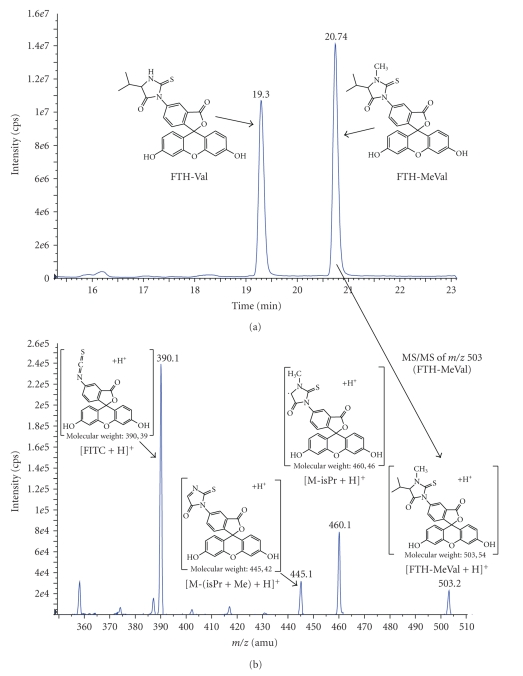
Expanded view of an LC-MS (TIC) chromatogram of standards **1** and **2** (a) and an LC-MS/MS spectrum of *m*/*z* = 503 (FTH-MeVal) (b).

Mass spectrometry was used for identification of the studied analytes and for measurement of their relative response under a number of selected conditions to optimise sensitivity. The measurement of LOD by LC/MS/MS was performed in order to compare the relative sensitivity between the analytes formed from the five isothiocyanate reagents studied, reacted with MeVal. Besides the N-methylated analytes (**2**, **4**, **6**–**8**) FTH-Val (**1**) was also included in order to study possible differences in LOD of the corresponding normal (unsubstituted) valine analyte as compared to FTH-MeVal. The LOD studies were performed by LC/MS system 1 after optimization of cone voltage and collision energy for the analytes at each given condition. The results from this system were confirmed and further improved with LC/MS/MS system no. 2. In this study all instrumental parameters were fully optimized which gave a general increase of the sensitivity by a factor of 25, when, for example, comparing the LOD's of FTH-MeVal by LC/MS system 1 with the LOD's by LC/MS/MS system 2 (obtained from values given in Tables 2 and 3). An example of the LC/MS/MS separation/analysis of FTH-Val and FTH-MeVal is presented in Figure 4, showing an expanded view of an LC-MS (TIC) chromatogram of the standards **1** and **2** and an LC-MS/MS spectrum of *m/z* = 503 (FTH-MeVal).

FTH-MeVal (**2**) gave the highest response on both systems compared to all other N-substituted analytes (**4**, **6, 7**) with one minor exception at one condition (DABTH-MeVal, unmodified condition, ESI+) (see Tables 2 and 3). Using the conditions stated for LC/MS system no. 1 the lowest LOD was 2.6 nM for FTH-MeVal under optimal conditions and 0.5 nM when measured with LC-system 2 (ESI, negative ions, ammonia).

The difference in LOD between FTH-Val and FTH-MeVal was measured in order to see the difference between N-methylated analytes (**2**) and normal valine analytes (**1**) (see Table 2). As this difference was relatively small, for example, as compared to the much larger difference between the five isothiocyanate reagents tested, further studies on normal valine analytes were excluded.

Initially the LODs were measured and compared for FTH-Val, FTH-MeVal, DABTH-MeVal, DNTH-MeVal, and PTH-MeVal (**1**, **2**, **4**, **6**, and **7**). The results from these studies are given in Tables 2 and 3. When it became clear that PTH-MeVal (**7**) gave a much lower relative response by LC/MS (ESI positive and negative ion modes) than the other compounds (**1**, **2**, **4**, and **6**), two additional analytes, PFPTH-MeVal (**8**) and PFPTH-HOEtVal (**9**), were also included in this study for a few comparative measurements. The results for compounds **8** and **9** gave an even lower relative response than those for PTH-MeVal (**7**); that is, PFPTH-MeVal (**8**) showed an LOD of 77,000 nM when measured with ESI (negative ions, unbuffered solvent system). An attempt to measure positive ions failed as product ions *m/z* 311 and *m/z* 266 were too weak to be measured. However, compound **8** could be measured when ionized with atmospheric pressure chemical ionization (APCI) and gave an LOD of 82,600 nM (0.1% TFA, H_2_O/ACN 1 : 1, v/v, negative ions measured) which was just above ten times lower compared to compound **9** (LOD = 950,000 nM) at this specific condition. When comparing the relative sensitivity between compounds **1**, **2**,** 4**, and **6 **(see Table 2) with compounds** 7**–**9**, at the same condition, the relative differences were very large, that is, 2,200 times lower LOD for compound **2** as compared with **7**, 18,000 times lower LOD for **2** as compared to **9**, and 210,000 times lower LOD for **2** as compared to **8**. PFPTH-HOEtVal (**9**) was also measured with APCI (buffered with NH_4_OAc, negative ions measured) which gave an LOD of 4,400 nM which can be compared to the LOD of 790 nM for compound **2** at this condition. 

### 3.6. Fluorescence

The fluorescence properties of FTH-Val (**1**) and FTH-MeVal (**2**) were measured by recording excitation and emission spectra. Measurements of the dianions of **1** and **2 **(Figure 3) which were obtained above pH 5, that is, at pH 7 and 9, gave maximum excitation wavelength of 492 nm and maximum emission at 515 nm with almost identical spectra for **1** and **2**; see Figure 2 recorded at pH 7. This means that the adduct, that is, the methyl group in FTH-MeVal, does not affect the spectroscopic properties. The relative fluorescence intensities of the dianions of **1** and **2** were around 360-fold higher as compared to the reference fluoranthene.

### 3.7. Capillary Electrophoresis (CE)

**Figure 5:**
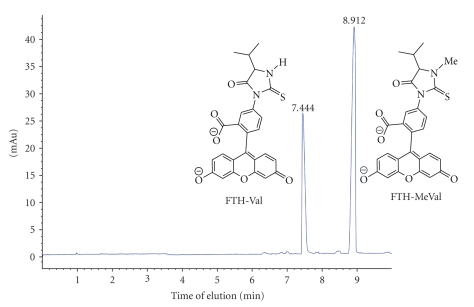
Separation of FTH-Val and FTH-MeVal by capillary electrophoresis and detection with a diode array.

The separation of FTH-MeVal from FTH-Val using a capillary electrophoresis-diode array detection (CE-DAD) system was evaluated in order to assess the possibility of determining N-terminal adducts using separation techniques other than LC as well as to be able to utilize and benefit from such sensitive techniques as capillary electrophoresis-laser-induced fluorescence detection (CE-LIF). This separation was performed using 17 mM phosphate buffer (adjusted to pH 7) containing 20 mM SDS. A complete baseline separation was obtained; FTH-Val eluted after 7.44 minutes and FTH MeVal after 8.91 minutes, both with narrow well-defined peaks (see Figure 5).

## 4. Discussion

### 4.1. 

Since the N-alkyl Edman-procedure was introduced 25 years ago [19] only small modifications of the originally presented procedure have been introduced. Initially, PITC was utilized as the Edman reagent. Somewhat later PFPITC was introduced, which gave a much higher sensitivity when measuring the analytes by GC/MS in the negative ion chemical ionization (NICI) mode [20]. The N-alkyl Edman-procedure using PITC or PFPITC has lately been adapted in order to perform the analysis by LC/MS/MS [10–14]. However, these modifications have so far not included studies of different Edman reagents to optimise the method for LC/MS analysis. The change from GC/MS to LC/MS has important implications for principles concerning separation and methods for the ionization of analytes.

The introduction of isothiocyanate reagents containing ionisable groups (i.e., FITC, DABITC, and DNITC) was demonstrated to be a valuable approach which gave several improvements, for example, increased solubility in blood at biological pH (regards FITC), and enhanced sensitivity when measuring by LC/MS/MS as compared to the PITC and PFPITC reagents (regards all three reagents). The ionic group and fluorescent properties of the analyte also provides new alternatives such as CE separation and spectroscopic detection, for example, with sensitive fluorescence detection. Ireland et al. showed, for example, that they could measure FTH-amino acids with a limit of detection on a low zeptomol (10^−21^) level per injection by using CE separation with laser induced fluorescence detection [21].

Although analysis with other methods for detection is possible when utilizing reagents such as FITC, MS/MS procedures are normally preferred due to the combination of high sensitivity, selectivity, and accuracy. This fundamental work was done in order to develop an LC/MS/MS procedure capable to deliver high sensitivity, throughput, and a method which is less restrictive regarding the types of adducts which can be measured.

### 4.2. Coupling and Detachment Reactions

One crucial question for the success of this procedure was if the coupling reaction as well as the detachment reaction (formation of the analytes already in the coupling reaction) would be hampered by the introduction of much bulkier reagents than used earlier. When using FITC, the largest Edman reagent tested, it was shown (e.g., on TLC) that N-substituted valines or N-substituted Val-Leu-anilides reacted readily and formed FTH-MeVal at the conditions for the coupling (pH ~ 8). The same result was obtained for DABITC forming DABTH-MeVal, and DNITC forming DNTH-MeVal. When thiocarbamoylating normal valine or ValLeuSer with FITC, DABITC and DNITC no rinclosure occurred as expected. To convert these products to thiohydantoin analytes an acidification step was demanded, which is in agreement with earlier studies with PITC and PFPITC [15, 17].

A bonus with FITC, DABITC, and DNITC is that the analytes formed are coloured. All steps could therefore easily be followed in real time by the naked eye, which was beneficial throughout the whole development work of the procedure as well as for preparation of synthetic standards.

### 4.3. LC-Separation Prior Analysis

All pairs of analytes, that is, FTH-Val and FTH-MeVal, **3**, and **4**, **5** and **6** could easily be separated with full baseline separation by using water/acetonitrile gradients on a standard reversed phase HPLC column. When using volatile pH modifiers such as 0.1% TFA, the solvent system is compatible with LC/MS/MS.

### 4.4. LC/MS/MS Analysis

In order to study and compare the relative sensitivity of the analytes (compounds **1**, **2**, **4**, **6**, and **7**) at selected conditions, negative and positive ions were measured by ESI, at acidic, neutral, and alkaline pH in H_2_O : ACN [1 : 1, (v/v)]. This experimental setup gave pH values both above and below *pK*_*a*_ of the analytes formed from FITC, DABITC, and DNITC. It was then surprisingly found that the relative response was the highest for the FTH analytes (**1** and **2**) under all tested conditions, with just one exception (DABTH-MeVal, no buffer, ESI+) (compare Tables 2 and 3). This high relative response obtained with the FTH analytes **1** and **2** was interpreted to be favoured by the readily ionisable groups (e.g., phenolic groups) in the fluorescein body, which can form both negative and positive ions. Another factor, which also increases the signal-to-noise ratio, is that the FTH-analytes had the highest molecular weights, which is beneficial as the chemical background noise decreases with higher *m/z*. The order of relative sensitivity for the studied analytes was (lowest LOD first); FTH-MeVal ~ FTH-Val > DABTH-MeVal > DNTH-MeVal > PTH-MeVal > PFPTH-HOEtVal (**9**) > PFPTH-MeVal (**8**). Interestingly the LODs for **9** were a factor 10 lower than for **8** which must be assigned to the hydroxyl group on **9 **which has higher ion affinity and is more prone to be ionized, giving lower LOD. This result indicates that the relative sensitivity of a specific analyte will vary more when PITC or PFPITC is used as the derivatisation reagent compared to, for example, FITC. For the PITC/PFPITC reagents the ion affinity of the adduct will determine the ion affinity in contrast to ionisable isothiocyanate reagents which are more prone to be ionised. Reagents such as FITC will therefore give less difference in obtained relative response as long as the ion affinity of the adduct is lower than the isothiocyanate body (under investigation).

The sensitivity obtained for especially the FITC reagent should be sufficient for measurement of background adduct levels of, for example, 30 pmol/g globin, common for ethylene oxide. From a sample containing 50 mg globin there will be 1.5 pmol ethylene oxide adduct. If this is quantitatively detached, purified, and concentrated to a volume of 100 *μ*L we will have a final concentration of 15 nM which is about 10 times over LOQ. Naturally there will be losses in the analytical chain and lower sensitivity with real samples due to, for example, ion suppression, but there will still be a good marginal to measure low background adduct levels. This marginal will also be enough for using TFA as pH modifier which gives better retention and sharper peaks when separated on a C_18_ LC column as the analytes then are neutral and much more lipophilic. Studies on real samples are ongoing and will be published soon.

This study was conducted on two different MS/MS systems to verify the rather drastic difference in sensitivity between the used Edman reagents seen on system no 1. Comparison of obtained relative sensitivity (LODs) of studied analytes gave rather constant ratios where system 2 gave 25 times lower LODs than system 1 (compare Table 3 with Table 2). It was expected that system 2 which is a triple quad instrument operating in mrm mode should give lower LODs than the LCQ iontrap instrument (system 1). This relative difference between the two systems could in fact be even higher as system 1 was used with direct injections of the analytes, giving no dilution at all, while system 2 used an LC column with a small i.d. (1.0 × 100 mm) operating at 50 *μ*L/min which definitively diluted the analytes as compared to direct injections. However, much effort was made to optimize system 2 to maximize the sensitivity. On more modern and sophisticated instruments we will expect even lower LODs and we will also expect that the relative difference between the analytes will be rather constant in accordance to this study. Finally, triple quad instruments such as system 2 operating in MRM mode mode are the instrument of choice for selectivity and sensitivity in quantitative measurements.

### 4.5. Capillary Electrophoresis and Fluorescence

Ireland et al. [21] showed that amino acids in proteins could be sequenced with FITC as Edman reagent and measured by CE-LIF with outstanding sensitivity. In order to test the same principle for adduct measurements without having access to a CE-LIF system we used CE-DAD to study the separation between FTH-Val and FTH-MeVal and measured the fluorescence properties separately. The conclusion of this study and Irelands work on FTH-amino acids is that the sensitivity of CE-LIF should be high enough for low adduct level measurements. A probable disadvantage with CE and fluorescence measurements is that by-products always are formed in huge excess when analyzing real samples. In spite of the high separation capacity of CE, by-products might interfere with the low adduct levels that are commonly found (pmol/g globin level) which corresponds to about one adduct per 1–10 million normal valines [22]. However, when measuring adducts levels that occurs at much higher levels such as aldehydes, CE separation with some kind of fluorescence measurement might be a valuable alternative to LC-MS/MS analysis. To exemplify this, Kautiainen et al. [23] recorded 1000 times higher levels of 2-hydroxyethylvaline (13–17 nmol/g globin) on reduced globin samples (probably source is the reduced Schiff base from acetaldehyde) as compared to levels between 16–23 pmol/g globin for unreduced samples (adducts formed directly from ethylene oxide). Finally, to combine powerful separation techniques (which are improved continuously), with fluorescence measurements and MS/MS analysis might be an interesting alternative for future work. As shown in this study, the spectroscopic properties were not affected by the adduct. The fluorescence properties could thus be used for quantification and MS/MS for structural characterisation. This combination of techniques would be powerful for analysis of today unknown adducts.

## 5. Conclusion

This study demonstrated that the N-alkyl Edman-procedure could be successfully adapted towards LC conditions by exchanging the isothiocyanate reagents with ionisable reagents such as FITC, DABITC, or DNITC. FITC was superior compared to the other reagents tested. The use of FITC as a reagent opens up for new possibilities for the analysis of haemoglobin adducts. The present study provides a basis for the development of the Adduct FI*R*E procedure [24] facilitating application of Hb adduct measurement in experimental and epidemiological studies.

## Figures and Tables

**Table 1:** Studied thiohydantoin analytes by LC/MS/MS.

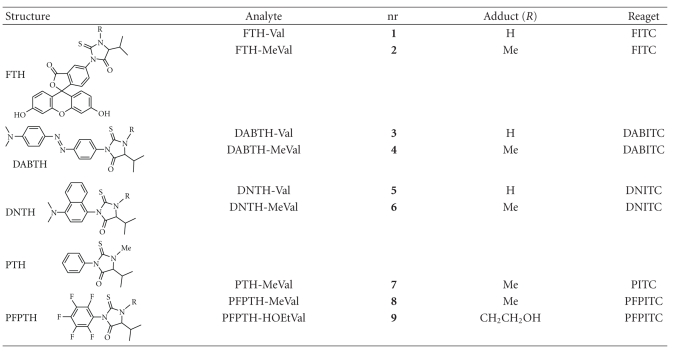

**Table 2:** Measurements, limit of detection (LOD) on pure standards, data obtained by LC/MS system no. 1.

Buffer/pH modifier (obtained pH) ionization method	TFA (~2) ESI	Without buffer/pH-modifier ESI		NH_4_OAc (~7) ESI		NH_3_ (~9) ESI		TFA (~2) APCI
	Positive	Positive	Negative	Positive	Negative	Positive	Negative	Positive
FTH-Val (LOD, nM)	15	5,200	750	47	21	61	10	8.4
FTH-MeVal (LOD, nM)	51	5,900	660	210	180	14	**2.7**	4.6
DABTH-MeVal (LOD, nM)	350	5,000	5,100	790	590	130	730	55
DNTH-MeVal (LOD, nM)	1,200	12,000	13,000	12,000	2800,000	6,100	210,000	110
PTH-MeVal (LOD, nM)	100,000	48,000	12,000	130,000	87,000	7,800	12,000	10,000

**Table 3:** Measurements, limit of detection (LOD) on pure standards by LC/MS/MS (ESI) with LC/MS system no. 2.

Buffer/pH modifier (obtained pH)	TFA (~2)	Without buffer/pH-modifier		NH_4_OAc (~7)		NH_3_ (~9)	
	Positive	Positive	Negative	Positive	Negative	Positive	Negative
FTH-MeVal (LOD, nM)	2.0	230	26	8.2	7.2	2.4	**0.5**
DABTH-MeVal (LOD, nM)	14	200	200	31	24	5.1	29
DNTH-MeVal (LOD, nM)	54	550	560	560	130,000	270	9,400
PTH-MeVal (LOD, nM)	4,100	19,000	470	7,500	3,400	310	470
